# Set-Membership Based Hybrid Kalman Filter for Nonlinear State Estimation under Systematic Uncertainty

**DOI:** 10.3390/s20030627

**Published:** 2020-01-22

**Authors:** Yan Zhao, Jing Zhang, Gaoge Hu, Yongmin Zhong

**Affiliations:** 1 Air and Missile Defense College, Air Force Engineering University, Xi’an 710051, China; zytyler@163.com; 2 School of Public Management, Xi’an University of Finance and Economics, Xi’an 710061, China; zhangjing@xaufe.edu.cn; 3 School of Automation, Northwestern Polytechnical University, Xi’an 710072, China; 4 School of Engineering, RMIT University, Bundoora, VIC 3083, Australia; yongmin.zhong@rmit.edu.au

**Keywords:** nonlinear state estimation, Kalman filtering, set-membership, systematic uncertainty, unknown but bounded error

## Abstract

This paper presents a new set-membership based hybrid Kalman filter (SM-HKF) by combining the Kalman filtering (KF) framework with the set-membership concept for nonlinear state estimation under systematic uncertainty consisted of both stochastic error and unknown but bounded (UBB) error. Upon the linearization of the nonlinear system model via a Taylor series expansion, this method introduces a new UBB error term by combining the linearization error with systematic UBB error through the Minkowski sum. Subsequently, an optimal Kalman gain is derived to minimize the mean squared error of the state estimate in the KF framework by taking both stochastic and UBB errors into account. The proposed SM-HKF handles the systematic UBB error, stochastic error as well as the linearization error simultaneously, thus overcoming the limitations of the extended Kalman filter (EKF). The effectiveness and superiority of the proposed SM-HKF have been verified through simulations and comparison analysis with EKF. It is shown that the SM-HKF outperforms EKF for nonlinear state estimation with systematic UBB error and stochastic error.

## 1. Introduction

The nonlinear state estimation problem has received significant attention in the fields of process control [1], tracking guidance [2], system identification [3], sensor networks [4], navigation [5,6] and so on. It is well known that the more accurate the system model is, the more accurate the state estimation can be obtained. However, due to the complexity of systematic dynamics and the dynamically changing conditions of the environment, uncertainty is inevitably involved in the system model [7]. This uncertainty is commonly characterized by stochastic errors such as the Gaussian or non-Gaussian system noise. With the assumption that the statistical characteristics of system noise are known, the issue of nonlinear system state estimation has been extensively studied [6,8,9,10]. However, in practical applications, the system uncertainty is mixed by stochastic errors as well as unknown but bounded (UBB) errors [11]. The UBB error, as implied by its name, refers to the systematic modelling uncertainty whose probability distribution is difficult to identify or even without the probabilistic nature. If the effect of UBB error on system dynamics is not considered in the filtering process, the state estimation will be biased or even diverged.

The Kalman filter (KF) is undoubtedly the most famous state estimation method within the Bayesian framework. As the system model is linear and the uncertainty is Gaussian distributed, KF is optimal in the sense of mean squared error [12]. Unfortunately, KF is only suitable for nominally linear systems due to its theoretical limitation. For nonlinear systems, a variant of KF, which is named as the extended Kalman filter (EKF), linearizes the nonlinear system model via a Taylor series expansion such that KF can be applied [13]. EKF has been used for many years as the benchmark in nonlinear state estimation. Some other candidates are the unscented Kalman filter (UKF) [5,6,14], cubature Kalman filter (CKF) [15] and particle filter (PF) [16]. However, since these nonlinear state estimation methods are based on the framework of KF, they also require the exact statistical knowledge of system uncertainty, which is difficult to achieve in engineering practice.

Research efforts have been devoted in improving the robustness of nonlinear filtering against unknown system uncertainty. These include the Huber’s M-Estimation based robust EKF [17], strong tracking UKF [7], covariance matching based adaptive UKF [18], Mahalanobis distance based robust CKF [19] and H-infinity based robust CKF [20], to name a few only. However, the existing techniques are mainly focused on the disturbances of the stochastic errors such as observation outliers, non-Gaussian noise and inaccurate noise statistics, rather than the disturbance of UBB error on nonlinear state estimation.

The set-membership filtering is a method to handle UBB error for nonlinear state estimation. The origination of the set-membership filtering can be dated back to the 1960s and such a problem has received recurring research interest in the past decade [11,21,22,23]. Different from the above nonlinear Kalman filters, the state estimation obtained by the set-membership filtering is a feasible set of possible states rather than a single value. This feasible set describes the range of the state estimate and guarantees that the estimation error is confined to a bounded region. Currently, the polytopes and ellipsoids are commonly used to describe the feasible set. Compared to the polytope method, which requires a great large number of inequalities to determine the feasible set, the ellipsoid method is more popular for online applications since it can represent the set with fewer pieces of information [21]. Therefore, for the sake of computational performance, the ellipsoid method is employed in this paper for feasible set description.

Among the existing studies on the set-membership filtering, the techniques reported in References [11] and [21] are quite constructive. In Reference [21], the concept of set-membership estimation was extended from a linear system to nonlinear system for the first time. An extended set-membership filter (ESMF) was also established via the linearization of the system model, in which the linearization error and system noise were combined into a new UBB noise term and was further addressed by use of the interval mathematics. However, this method does not take the substantive UBB errors of a system into account and the combination of the linearization error and system noise may degrade the state estimation accuracy since the statistical knowledge of system noise is omitted. In Reference [11], considering both UBB error and stochastic error in the Kalman filtering scheme, a new Kalman gain was obtained by minimizing the mean squared error of the system state based on set-membership. However, the technique presented in Reference [11] is only suitable for linear systems.

Motivated by the techniques reported in References [11] and [21], this paper proposes a set-membership based hybrid Kalman filter (SM-HKF) for nonlinear system state estimation in the presence of both UBB error and stochastic error. This method linearizes the nonlinear system model by a Taylor series expansion and then combines the linearization error with the systematic UBB error to generate a new UBB error term through the Minkowski sum. Further, it derives an optimal Kalman gain under the criterion of minimum mean squared error based on the framework of KF. The proposed SM-HKF extends the set-membership based Kalman filter in Reference [11] from linear systems to nonlinear systems. It also overcomes the limitation of EKF by treating the systematic UBB error, stochastic error and linearization error simultaneously. Simulations and comparison analysis have been conducted to evaluate the effectiveness of the proposed method.

## 2. Definitions on Ellipsoidal Sets

To facilitate the problem formulation and the derivation of SM-HKF, several definitions for describing ellipsoidal sets are introduced for notational convenience.

**Definition** **1.** *An ellipsoid*ξ(a,P)*is given by the set*

(1) $$\xi \left(a,P\right)=\left\{x\in {\mathbb{R}}^{n}:{\left(x-a\right)}^{T}{\left(P\right)}_{}^{-1}\left(x-a\right)\leq 1\right\},$$

*where*a*is the center of the ellipsoid,*P*is symmetric positive definite shape matrix and*x is any point within the ellipsoid.

**Definition** **2.** *The affine transition of the ellipsoid set can be computed as*

(2) $$A\xi \left(a,P\right)=\xi \left(Aa,APA^{T}\right),$$

*where* A *is a parameterized matrix.*

**Definition** **3.** *Supposing* $\xi \left(a_{1},P_{1}\right)$ *and* $\xi \left(a_{2},P_{2}\right)$ *are two ellipsoidal sets, the Minkowski sum of the ellipsoids is defined as*

(3) $$S_{S}=\left\{x:\;x=x_{1}+x_{2},\;x_{1}\in \xi \left(a_{1},P_{1}\right),x_{2}\in \xi \left(a_{2},P_{2}\right)\right\}$$

*and denoted by*

(4) $$S_{S}=\xi \left(a_{1},P_{1}\right)⊕\xi \left(a_{2},P_{2}\right).$$

Generally, the set $S_{S}$ is not an ellipsoid. We can find an outer bounding ellipsoid $\xi_{S}\left(a^{\prime },P^{\prime }(\rho )\right)$ such that

(5) $$S_{S}\subseteq \xi_{S}\left(a^{\prime },P^{\prime }(\rho )\right).$$

That is,

(6) $$\xi \left(a_{1},P_{1}\right)⊕\xi \left(a_{2},P_{2}\right)\subseteq \xi_{S}\left(a^{\prime },P^{\prime }(\rho )\right),$$

with

(7) $$a^{\prime }=a_{1}+a_{2}$$

(8) $$P^{\prime }(\rho )=\left(1+\rho^{-1}\right)P_{1}+\left(1+\rho \right)P_{2},$$

where ρ∈(0,1) defines the weights on $P_{1}$ and $P_{2}$, which can be determined by minimizing the semi-axes of the ellipsoid, that is [11]

(9) $$\rho =trace{\left(P_{1}\right)}^{\frac{1}{2}}\cdot trace{\left(P_{2}\right)}^{-\frac{1}{2}}.$$

Some other alternatives for computing the value of ρ can be found in Reference [24].

## 3. Set-Membership Based Hybrid Kalman Filter

### 3.1. Problem Formulation

Consider the following nonlinear discrete-time dynamical system

(10) $$x_{k}=f\left(x_{k-1}\right)+\delta_{k}^{xU}+\delta_{k}^{xG}$$

(11) $$z_{k}=h\left(x_{k}\right)+\delta_{k}^{yU}+\delta_{k}^{yG},$$

where $x_{k}\in {\mathbb{R}}^{n}$ and $z_{k}\in {\mathbb{R}}^{m}$, both f(⋅) and h(⋅) are assumed to be twice continuously differentiable functions; $\delta_{k}^{xG}\in {\mathbb{R}}^{n}$ and $\delta_{k}^{yG}\in {\mathbb{R}}^{m}$ are the zero-mean Gaussian white noises with covariance matrices $Q_{k}^{xG}$ and $R_{k}^{yG}$; $\delta_{k}^{xU}\in \xi \left(0_{n\times 1},Q_{k}^{xU}\right)$ and $\delta_{k}^{yU}\in \xi \left(0_{m\times 1},R_{k}^{yU}\right)$ are the UBB errors involved in the process model and measurement model; and $\delta_{k}^{xG}$, $\delta_{k}^{yG}$, $\delta_{k}^{xU}$ and $\delta_{k}^{yU}$ are considered to be mutual independent.

Expanding (10) by a Taylor series about the system state estimate ${\hat{x}}_{k-1}$, we have

(12) $$x_{k}=f\left({\hat{x}}_{k-1}\right)+\frac{\partial f\left(x_{k-1}\right)}{\partial x_{k-1}}|{}_{x_{k-1}={\hat{x}}_{k-1}}\left(x_{k-1}-{\hat{x}}_{k-1}\right)+o_{f}\left(x_{k-1}-{\hat{x}}_{k-1}\right)+\delta_{k-1}^{xG}+\delta_{k-1}^{xU},$$

where $o_{f}\left(x_{k-1}-{\hat{x}}_{k-1}\right)$ denotes the higher-order remainder term in the Taylor series.

The interval mathematics is used to bound the linearization error $o_{f}\left(x_{k-1}-{\hat{x}}_{k-1}\right)$. Suppose the ellipsoidal sets of the system state at time k−1 is $\xi \left({\hat{x}}_{k-1},{\hat{P}}_{k-1}\right)$. The extrema of this state ellipsoid are computed as

(13) $${\hat{x}}_{k-1,-}^{i}={\hat{x}}_{k-1}^{i}-\sqrt{{\hat{P}}_{k-1}^{i,i}}\;\mathrm{and}\;{\hat{x}}_{k-1,+}^{i}={\hat{x}}_{k-1}^{i}+\sqrt{{\hat{P}}_{k-1}^{i,i}}\;(i=1,2,\cdots ,n),$$

where ${\hat{x}}_{k-1}^{i}$ is the ith component of ${\hat{x}}_{k-1}^{}$; the subscripts “−” and “+” denote the minimum and maximum values; and ${\hat{P}}_{k-1}^{i,i}$ is the ith diagonal element of ${\hat{P}}_{k-1}$.

The state interval bound $X_{k-1}^{}$ for ${\hat{x}}_{k-1}^{}$ is then defined as

(14) $$X_{k-1}^{i}=\left[{\hat{x}}_{k-1,-}^{i},{\hat{x}}_{k-1,+}^{i}\right]\;(i=1,2,\cdots ,n)$$

and the interval for the linearization error can be further determined by

(15) $$X_{R}^{}(k-1)=diag\{X_{k-1}^{T}\}\left[\begin{array}{c} Hes_{1}\\ Hes_{2}\\ \vdots \\ Hes_{n} \end{array}\right]X_{k-1}^{},$$

where $Hes_{i}$(i=1,2,⋯,n) represents the Hessian matrix of f(⋅) at $X_{k-1}^{}$.

Based on the results in Reference [21], the interval $X_{R}^{}(k-1)$ can be bounded using an outer bounding ellipsoid $\xi \left(0_{n\times 1},{\bar{Q}}_{k}^{x}\right)$, in which

(16) $${\left({\bar{Q}}_{k}^{x}\right)}^{i,i}=2{\left[{\left(X_{R}^{}(k-1)\right)}^{i}\right]}^{2}$$

(17) $${\left({\bar{Q}}_{k}^{x}\right)}^{i,j}=0_{n\times 1}\;(i\neq j).$$

After that, we denote the linearization error as $\delta_{k}^{xL}=o_{f}\left(x_{k-1}-{\hat{x}}_{k-1}\right)$. By taking the linearization error into account, the process model (10) can be rewritten in the following linear form

(18) $$x_{k}=f\left({\hat{x}}_{k-1}\right)+F_{k}\left(x_{k-1}-{\hat{x}}_{k-1}\right)+\delta_{k}^{xL}+\delta_{k}^{xU}+\delta_{k}^{xG},$$

where $F_{k}=\frac{\partial f\left(x_{k-1}\right)}{\partial x_{k-1}}|{}_{x_{k-1}={\hat{x}}_{k-1}}$ and $\delta_{k}^{xL}\in \xi \left(0_{n\times 1},{\bar{Q}}_{k}^{x}\right)$.

Similar to (12)–(18), an outer bounding ellipsoid $\xi \left(0_{m\times 1},{\bar{R}}_{k}^{y}\right)$ is easy to achieve to bound the linearization error of h(⋅) such that the measurement model (11) can be rewritten in the following linear form

(19) $$z_{k}=h\left({\hat{x}}_{k/k-1}\right)+H_{k}(x_{k}-{\hat{x}}_{k/k-1})+\delta_{k}^{yL}+\delta_{k}^{yU}+\delta_{k}^{yG},$$

where ${\hat{x}}_{k/k-1}=f\left({\hat{x}}_{k-1}\right)$ denotes the predicted state estimate, $H_{k}=\frac{\partial h\left(x_{k}\right)}{\partial x_{k}}|{}_{x_{k}={\hat{x}}_{k/k-1}}$ and $\delta_{k}^{yL}\in \xi \left(0_{m\times 1},{\bar{R}}_{k}^{y}\right)$ is the linearization error.

For the dynamic system described by (18) and (19), we shall discuss in the following how to estimate the system state in the presence of both UBB and stochastic errors.

### 3.2. Optimal Kalman Gain for Nonlinear System with UBB Error and Stochastic Error

Since the linearization error is also unknown but bounded, we firstly combine it with the systematic UBB error to generate a new UBB error term through the Minkowski sum.

For the process model given by (18), by summing the linearization error $\delta_{k}^{xL}$ and the systematic UBB error $\delta_{k}^{xU}$, a new UBB error ${\bar{\delta }}_{k}^{xU}$ can be defined as

(20) $${\bar{\delta }}_{k}^{xU}\in \xi \left(0_{n\times 1},{\bar{Q}}_{k}^{xU}\right)=\xi \left(0_{n\times 1},{\bar{Q}}_{k}^{x}\right)⊕\xi \left(0_{n\times 1},Q_{k}^{xU}\right).$$

Similarly, for the measurement model given by (19), another new UBB error ${\bar{\delta }}_{k}^{yU}$ is introduced by the sum of $\delta_{k}^{yL}$ and $\delta_{k}^{yU}$

(21) $${\bar{\delta }}_{k}^{yU}\in \xi \left(0_{m\times 1},{\bar{R}}_{k}^{xU}\right)=\xi \left(0_{m\times 1},{\bar{R}}_{k}^{y}\right)⊕\xi \left(0_{m\times 1},R_{k}^{yU}\right).$$

Then, the dynamic system described by (18) and (19) can be further rewritten as

(22) $$x_{k}=f\left({\hat{x}}_{k-1}\right)+F_{k}\left(x_{k-1}-{\hat{x}}_{k-1}\right)+{\bar{\delta }}_{k}^{xU}+\delta_{k}^{xG}$$

(23) $$z_{k}=h\left({\hat{x}}_{k/k-1}\right)+H_{k}(x_{k}-{\hat{x}}_{k/k-1})+{\bar{\delta }}_{k}^{yU}+\delta_{k}^{yG},$$

where $\delta_{k}^{xG}$, $\delta_{k}^{yG}$, ${\bar{\delta }}_{k}^{xU}$ and ${\bar{\delta }}_{k}^{yU}$ are independent of each other.

In the following, we shall derive the optimal Kalman gain based on the system described by (22) and (23).

Suppose the system state estimate at time k−1 is ${\hat{x}}_{k-1}$, whose error covariance is ${\hat{P}}_{k-1}$. From the KF framework, the state estimate at time k can be obtained by

(24) $${\hat{x}}_{k}=f\left({\hat{x}}_{k-1}\right)+K_{k}\left(z_{k}-h\left({\hat{x}}_{k/k-1}\right)\right),$$

where $K_{k}$ is the Kalman gain that we are looking for to minimize the mean squared error of the state estimate.

To simplify the vector operations, we employ the notation ${\left(x\right)}^{2}=x\cdot x^{T}$ for any vector x throughout this paper. Since $x_{k-1}-{\hat{x}}_{k-1}$ is uncorrelated to $\delta_{k}^{xG}$, $\delta_{k}^{yG}$, ${\bar{\delta }}_{k}^{xU}$ and ${\bar{\delta }}_{k}^{yU}$, it is verified from (22)~(24) that the error covariance of ${\hat{x}}_{k}$ can be expressed as

(25) $$\begin{array}{ll} & E\left\{{\left({\hat{x}}_{k}^{}-x_{k}^{}\right)}^{2}\right\}\\ = & E\left\{{\left[\left(f\left({\hat{x}}_{k-1}\right)+K_{k}\left(z_{k}-h\left({\hat{x}}_{k/k-1}\right)\right)\right)-\left(f\left({\hat{x}}_{k-1}\right)+F_{k}\left(x_{k-1}-{\hat{x}}_{k-1}\right)+{\bar{\delta }}_{k}^{xU}+\delta_{k}^{xG}\right)\right]}^{2}\right\}\\ = & E\left\{{\left[\left(K_{k}\left(H_{k}(x_{k}-{\hat{x}}_{k/k-1})+{\bar{\delta }}_{k}^{yU}+\delta_{k}^{yG}\right)\right)-\left(F_{k}\left(x_{k-1}-{\hat{x}}_{k-1}\right)+{\bar{\delta }}_{k}^{xU}+\delta_{k}^{xG}\right)\right]}^{2}\right\}\\ = & E\left\{{\left[\left(K_{k}\left(H_{k}\left(F_{k}\left(x_{k-1}-{\hat{x}}_{k-1}\right)+{\bar{\delta }}_{k}^{xU}+\delta_{k}^{xG}\right)+{\bar{\delta }}_{k}^{yU}+\delta_{k}^{yG}\right)\right)-\left(F_{k}\left(x_{k-1}-{\hat{x}}_{k-1}\right)+{\bar{\delta }}_{k}^{xU}+\delta_{k}^{xG}\right)\right]}^{2}\right\}\\ = & E\left\{{\left[\left(K_{k}H_{k}-Ι\right)F_{k}\left(x_{k-1}-{\hat{x}}_{k-1}\right)+\left(K_{k}H_{k}-Ι\right){\bar{\delta }}_{k}^{xU}+\left(K_{k}H_{k}-Ι\right)\delta_{k}^{xG}+K_{k}{\bar{\delta }}_{k}^{yU}+K_{k}\delta_{k}^{yG}\right]}^{2}\right\}\\ = & \left(K_{k}H_{k}-Ι\right)\left(F_{k}{\hat{P}}_{k-1}F_{k}^{T}+Q_{k}^{xG}\right){\left(K_{k}H_{k}-Ι\right)}^{T}+K_{k}R_{k}^{yG}K_{k}^{T}+{\left(\left(K_{k}H_{k}-Ι\right){\bar{\delta }}_{k}^{xU}+K_{k}{\bar{\delta }}_{k}^{yU}\right)}^{2} \end{array}$$

Due to the set-membership of ${\bar{\delta }}_{k}^{xU}$ and ${\bar{\delta }}_{k}^{yU}$ as shown in (20) and (21), the last term in (25) can be computed as the Minkowki sum, that is,

(26) $$\begin{array}{ll} & \left(K_{k}H_{k}-Ι\right){\bar{\delta }}_{k}^{xU}+K_{k}{\bar{\delta }}_{k}^{yU}\\ \in & \left(K_{k}H_{k}-Ι\right)\xi \left(0_{n\times 1},{\bar{Q}}_{k}^{xU}\right)⊕K_{k}\xi \left(0_{m\times 1},{\bar{R}}_{k}^{xU}\right)\\ = & \xi \left(0_{n\times 1},\left(K_{k}H_{k}-Ι\right){\bar{Q}}_{k}^{xU}{\left(K_{k}H_{k}-Ι\right)}^{T}\right)⊕\xi \left(0_{n\times 1},K_{k}{\bar{R}}_{k}^{xU}K_{k}^{T}\right)\\ \subset & \xi \left(0_{n\times 1},\varpi (\rho )\right) \end{array}$$

in which

(27) $$\varpi (\rho )=\left(1+\rho^{-1}\right)\left(K_{k}H_{k}-Ι\right){\bar{Q}}_{k}^{xU}{\left(K_{k}H_{k}-Ι\right)}^{T}+\left(1+\rho \right)K_{k}{\bar{R}}_{k}^{xU}K_{k}^{T}$$

where ρ∈(0,1) is determined according to (9).

Then, from (25) and (26), the trace of $E\left\{{\left({\hat{x}}_{k}-x_{k}\right)}^{2}\right\}$ can be computed by

(28) $$\begin{array}{ll} & trace\left(E\left\{{\left({\hat{x}}_{k}^{}-x_{k}^{}\right)}^{2}\right\}\right)\\ = & trace\left(\left(K_{k}H_{k}-Ι\right)\left(F_{k}{\hat{P}}_{k-1}F_{k}^{T}+Q_{k}^{xG}\right){\left(K_{k}H_{k}-Ι\right)}^{T}\right)+trace\left(K_{k}R_{k}^{yG}K_{k}^{T}\right)\\ & +trace\left({\left(\left(K_{k}H_{k}-Ι\right){\bar{\delta }}_{k}^{xU}+K_{k}{\bar{\delta }}_{k}^{yU}\right)}^{2}\right)\\ \leq & trace\left(\left(K_{k}H_{k}-Ι\right)\left(F_{k}{\hat{P}}_{k-1}F_{k}^{T}+Q_{k}^{xG}\right){\left(K_{k}H_{k}-Ι\right)}^{T}\right)+trace\left(K_{k}R_{k}^{yG}K_{k}^{T}\right)+trace\left(\varpi (\rho )\right)\\ = & trace\left(\left(K_{k}H_{k}-Ι\right)\left(F_{k}{\hat{P}}_{k-1}F_{k}^{T}+Q_{k}^{xG}\right){\left(K_{k}H_{k}-Ι\right)}^{T}\right)+trace\left(K_{k}R_{k}^{yG}K_{k}^{T}\right)\\ & +trace\left(\left(1+\rho^{-1}\right)\left(K_{k}H_{k}-Ι\right){\bar{Q}}_{k}^{xU}{\left(K_{k}H_{k}-Ι\right)}^{T}\right)+trace\left(\left(1+\rho \right)K_{k}{\bar{R}}_{k}^{xU}K_{k}^{T}\right) \end{array}$$

In order to determine the optimal Kalman gain $K_{k}$ which minimizes the error covariance of ${\hat{x}}_{k}^{}$, the condition $\frac{\partial }{\partial K_{k}}\left(trace\left(E\left\{{\left({\hat{x}}_{k}^{}-x_{k}^{}\right)}^{2}\right\}\right)\right)=0$ has to be fulfilled. By use of the derivative rules for the trace, we obtain the derivations of the first and second terms of (28) as follows

(29) $$\begin{array}{ll} & \frac{\partial }{\partial K_{k}}\left(trace\left(\left(K_{k}H_{k}-Ι\right)\left(F_{k}{\hat{P}}_{k-1}F_{k}^{T}+Q_{k}^{xG}\right){\left(K_{k}H_{k}-Ι\right)}^{T}\right)\right)\\ = & -\frac{\partial }{\partial K_{k}}\left(trace\left(K_{k}H_{k}\left(F_{k}{\hat{P}}_{k-1}F_{k}^{T}+Q_{k}^{xG}\right)\right)\right)-\frac{\partial }{\partial K_{k}}\left(trace\left(\left(F_{k}{\hat{P}}_{k-1}F_{k}^{T}+Q_{k}^{xG}\right)H_{k}^{T}K_{k}^{T}\right)\right)\\ & +\frac{\partial }{\partial K_{k}}\left(trace\left(K_{k}H_{k}\left(F_{k}{\hat{P}}_{k-1}F_{k}^{T}+Q_{k}^{xG}\right)H_{k}^{T}K_{k}^{T}\right)\right)+\frac{\partial }{\partial K_{k}}\left(trace\left(F_{k}{\hat{P}}_{k-1}F_{k}^{T}+Q_{k}^{xG}\right)\right)\\ = & -2\left(F_{k}{\hat{P}}_{k-1}F_{k}^{T}+Q_{k}^{xG}\right)H_{k}^{T}+2K_{k}H_{k}\left(F_{k}{\hat{P}}_{k-1}F_{k}^{T}+Q_{k}^{xG}\right)H_{k}^{T} \end{array}$$

and

(30) $$\frac{\partial }{\partial K_{k}}\left(trace\left(K_{k}R_{k}^{yG}K_{k}^{T}\right)\right)=2K_{k}R_{k}^{yG}$$

Similar to (29) and (30), the derivations of the third and fourth terms of (28) can be readily given by

(31) $$\frac{\partial }{\partial K_{k}}\left(trace\left(\left(1+\rho^{-1}\right)\left(K_{k}H_{k}-Ι\right){\bar{Q}}_{k}^{xU}{\left(K_{k}H_{k}-Ι\right)}^{T}\right)\right)=2\left(1+\rho^{-1}\right)\left(-{\bar{Q}}_{k}^{xU}H_{k}^{T}+K_{k}H_{k}{\bar{Q}}_{k}^{xU}H_{k}^{T}\right)$$

and

(32) $$\frac{\partial }{\partial K_{k}}\left(trace\left(\left(1+\rho \right)K_{k}{\bar{R}}_{k}^{xU}K_{k}^{T}\right)\right)=2\left(1+\rho \right)K_{k}{\bar{R}}_{k}^{xU}.$$

Thus, substituting (29)–(32) into the Equation $\frac{\partial }{\partial K_{k}}\left(trace\left(E\left\{{\left({\hat{x}}_{k}^{}-x_{k}^{}\right)}^{2}\right\}\right)\right)=0$, the optimal Kalman gain is yielded

(33) $$\begin{array}{ll} K_{k}= & \left(\left(F_{k}{\hat{P}}_{k-1}F_{k}^{T}+Q_{k}^{xG}\right)H_{k}^{T}+\left(1+\rho^{-1}\right){\bar{Q}}_{k}^{xU}H_{k}^{T}\right)\\ & \cdot {\left(\left(1+\rho^{-1}\right)H_{k}{\bar{Q}}_{k}^{xU}H_{k}^{T}+\left(1+\rho \right){\bar{R}}_{k}^{xU}+H_{k}\left(F_{k}{\hat{P}}_{k-1}F_{k}^{T}+Q_{k}^{xG}\right)H_{k}^{T}+R_{k}^{yG}\right)}^{-1} \end{array}.$$

It should be noted that, by simultaneous treatment of the systematic UBB error, stochastic error and linearization error, the SM-HKF established based on the Kalman Gain $K_{k}$ in (33) can restrain the effects of both UBB error and stochastic error on nonlinear state estimation in a hybrid way, which makes the robust filtering a reality.

Further, with the obtained optimal Kalman gain, the covariance matrix of ${\hat{x}}_{k}$ can be readily obtained by substituting (26) and (27) into (25)

(34) $$\begin{array}{ll} {\hat{P}}_{k} & =E\left\{{\left({\hat{x}}_{k}^{}-x_{k}^{}\right)}^{2}\right\}\\ & =\left(K_{k}H_{k}-Ι\right)\left(F_{k}{\hat{P}}_{k-1}F_{k}^{T}+Q_{k}^{xG}\right){\left(K_{k}H_{k}-Ι\right)}^{T}+K_{k}R_{k}^{yG}K_{k}^{T}+E\left\{{\left(\left(K_{k}H_{k}-Ι\right){\bar{\delta }}_{k}^{xU}+K_{k}{\bar{\delta }}_{k}^{yU}\right)}^{2}\right\}\\ & \leq \left(K_{k}H_{k}-Ι\right)\left(F_{k}{\hat{P}}_{k-1}F_{k}^{T}+Q_{k}^{xG}\right){\left(K_{k}H_{k}-Ι\right)}^{T}+K_{k}R_{k}^{yG}K_{k}^{T}\\ & \;+\left(1+\rho^{-1}\right)\left(K_{k}H_{k}-Ι\right){\bar{Q}}_{k}^{xU}{\left(K_{k}H_{k}-Ι\right)}^{T}+\left(1+\rho \right)K_{k}{\bar{R}}_{k}^{xU}K_{k}^{T} \end{array}$$

where ρ∈(0,1).

**Remark** **1.** *Suppose the dynamic system described by (10) and (11) does not involve UBB error and the linearization error is neglected, the Kalman Gain* $K_{k}$ *given in (33) can be simplified as*

(35) $$K_{k}=\left(\left(F_{k}{\hat{P}}_{k-1}F_{k}^{T}+Q_{k}^{xG}\right)H_{k}^{T}\right)\cdot {\left(H_{k}\left(F_{k}{\hat{P}}_{k-1}F_{k}^{T}+Q_{k}^{xG}\right)H_{k}^{T}+R_{k}^{yG}\right)}^{-1},$$

*which is exactly the Kalman Gain in EKF.*

**Remark** **2.** *In the KF framework, the calculation of the predicted state and its covariance matrix is actually an important intermediate step to achieve the ultimate state estimation, even though we do not display it particularly in the derivation of the optimal Kalman gain. Denote the predicted state as*${\hat{x}}_{k/k-1}=f\left({\hat{x}}_{k-1}\right)$*. From (22), the covariance matrix of*${\hat{x}}_{k/k-1}$*can be represented as*

(36) $$\begin{array}{l} {\hat{P}}_{k/k-1}=E\left\{{\left({\hat{x}}_{k/k-1}-x_{k}^{}\right)}^{2}\right\}\\ \;=E\left\{{\left[f\left({\hat{x}}_{k-1}\right)-\left(f\left({\hat{x}}_{k-1}\right)+F_{k}\left(x_{k-1}-{\hat{x}}_{k-1}\right)+{\bar{\delta }}_{k}^{xU}+\delta_{k}^{xG}\right)\right]}^{2}\right\}\\ \;=E\left\{{\left[\left(F_{k}\left(x_{k-1}-{\hat{x}}_{k-1}\right)+{\bar{\delta }}_{k}^{xU}+\delta_{k}^{xG}\right)\right]}^{2}\right\}\\ \;=F_{k}{\hat{P}}_{k-1}F_{k}^{T}+Q_{k}^{xG}+E\left\{{\left({\bar{\delta }}_{k}^{xU}\right)}^{2}\right\}\\ \;\leq F_{k}{\hat{P}}_{k-1}F_{k}^{T}+Q_{k}^{xG}+{\bar{Q}}_{k}^{xU} \end{array}$$

*where the result of (20) is employed*.

**Remark** **3.** *When using (35) and (36) in practical engineering,* ${\hat{P}}_{k/k-1}$ *and* ${\hat{P}}_{k}$ *are commonly calculated by*

(37) $${\hat{P}}_{k/k-1}=F_{k}{\hat{P}}_{k-1}F_{k}^{T}+Q_{k}^{xG}+{\bar{Q}}_{k}^{xU}$$

(38) $$\begin{array}{ll} {\hat{P}}_{k} & =\left(K_{k}H_{k}-Ι\right)\left(F_{k}{\hat{P}}_{k-1}F_{k}^{T}+Q_{k}^{xG}\right){\left(K_{k}H_{k}-Ι\right)}^{T}+K_{k}R_{k}^{yG}K_{k}^{T}\\ & +\left(1+\rho^{-1}\right)\left(K_{k}H_{k}-Ι\right){\bar{Q}}_{k}^{xU}{\left(K_{k}H_{k}-Ι\right)}^{T}+\left(1+\rho \right)K_{k}{\bar{R}}_{k}^{xU}K_{k}^{T} \end{array}.$$

*Although (37) and (38) may be of some conservatism, the usage of them can improve the convergence speed of SM-HKF, which is similar to the traditional Kalman filter.*

### 3.3. The SM-HKF Algorithm

Considering the nonlinear discrete-time dynamic system described by (10) and (11) and their equivalent equations (22) and (23), the proposed SM-HKF algorithm can be summarized as follows.

**Step 1**. Give the state estimate ${\hat{x}}_{k-1}$ and its error covariance ${\hat{P}}_{k-1}$.

**Step 2.** Calculate the predicted state estimate by ${\hat{x}}_{k/k-1}=f\left({\hat{x}}_{k-1}\right)$.

**Step 3.** Compute the optimal Kalman gain given by (33), in which (20) and (21) are used.

**Step 4.** Calculate the state estimation ${\hat{x}}_{k}$ and its covariance matrix ${\hat{P}}_{k}$ by (24) and (38), respectively.

**Step 5.** Repeat Steps 1 to 4 for the next time step.

## 4. Performance Evaluation

Simulations have been conducted to evaluate the performance of the proposed SM-HKF in the presence of UBB error and stochastic error in comparison with EKF. A two-dimensional target tracking model is considered, in which the state vector is composed of the vehicle position and velocity in East and North while the measurement vector the azimuth angle and slope distance of the vehicle.

Define the system state as $x_{k}={\left[\begin{array}{cccc} s_{E} & s_{N} & v_{E} & v_{N} \end{array}\right]}^{T}$, the process model and measurement model are given by

(39) $$x_{k}=f\left(x_{k-1}\right)+\delta_{k}^{xU}+\delta_{k}^{xG}$$

(40) $$z_{k}=h\left(x_{k}\right)+\delta_{k}^{yU}+\delta_{k}^{yG},$$

where $\delta_{k}^{xG}$ and $\delta_{k}^{yG}$ are the zero-mean Gaussian white noises (i.e., stochastic errors); $\delta_{k}^{xU}$ and $\delta_{k}^{yU}$ are the UBB errors; and

(41) $$f\left(x_{k-1}\right)=\left[\begin{array}{cccc} 1 & 0 & t & 0\\ 0 & 1 & 0 & t\\ 0 & 0 & 1 & 0\\ 0 & 0 & 0 & 1 \end{array}\right]x_{k-1}$$

(42) $$h\left(x_{k}\right)=\left[\begin{array}{c} \sqrt{s_{E}{}^{2}+s_{N}{}^{2}}\\ \arctan\left(s_{N}/s_{E}\right) \end{array}\right]$$

The covariance matrices of the Gaussian white noises in the process and measurement models are assumed to be

(43) QkxG=[t3300t22000t3300t220t2200t1000t2200t10]

(44) RkzG=[0.3210000.1210]

where the constant t is the sampling interval.

The shape matrix of the bounding ellipsoid describing the UBB error in the process model is

(45) QkxU=[axt330axt2200ayt330ayt22axt220axt00ayt220ayt]

where $a_{x}$ and $a_{x}$ are the accelerations in East and North introduced in the vehicle trajectory simulation.

Commonly, the measurement model can be established exquisitely based on the prior physical characteristics of measurement device and its accuracy can be further improved using a large amount of available measurement data [7]. Thus, we assume that there exist no UBB error in the measurement model which is given by

(46) $$R_{k}^{yU}=0$$

**Figure 1:**
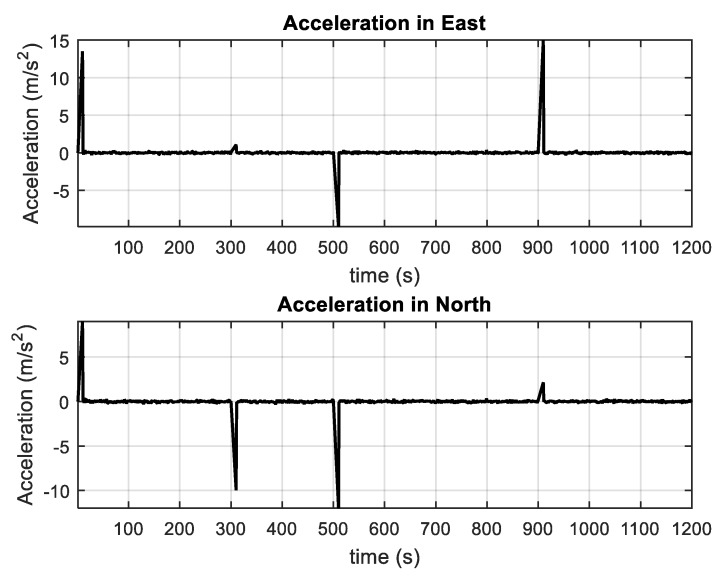
The accelerations involved in the simulated vehicle trajectory.

**Figure 2:**
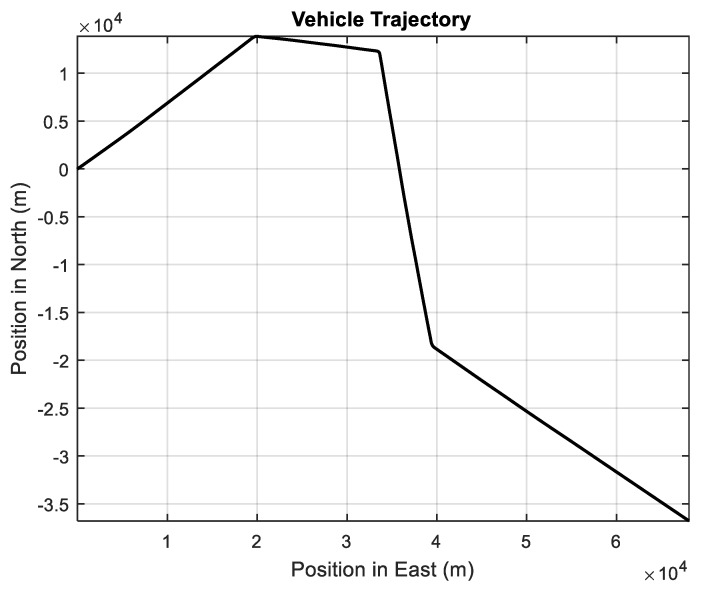
The vehicle trajectory.

The vehicle accelerations in East and North as well as the simulated vehicle trajectory are shown in Figure 1 and Figure 2. The vehicle acceleration variations are presented in Table 1, where the increases and decreases of the vehicle accelerations in East and North are described by UBB error and the stochastic fluctuation is described by Gaussian white noise in the process model. The simulation time is 1200 s and the sampling interval is 1 s.

**Figure 3:**
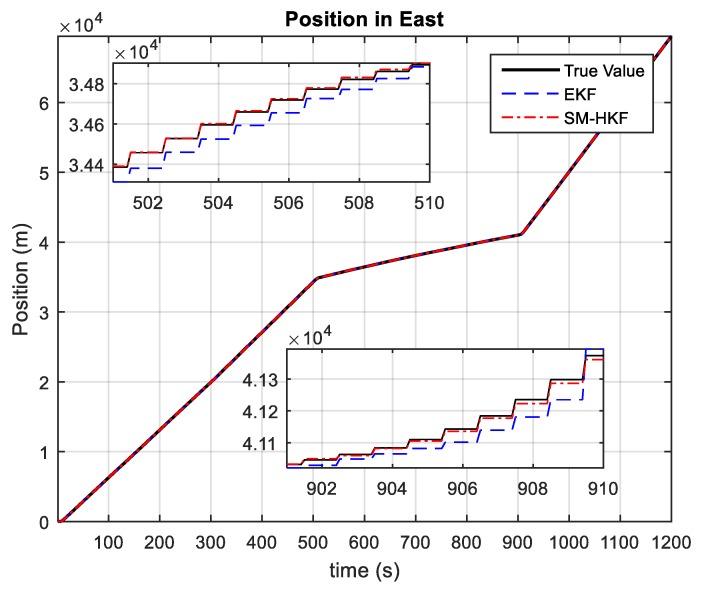
The positions in East estimated by EKF and SM-HKF.

**Figure 4:**
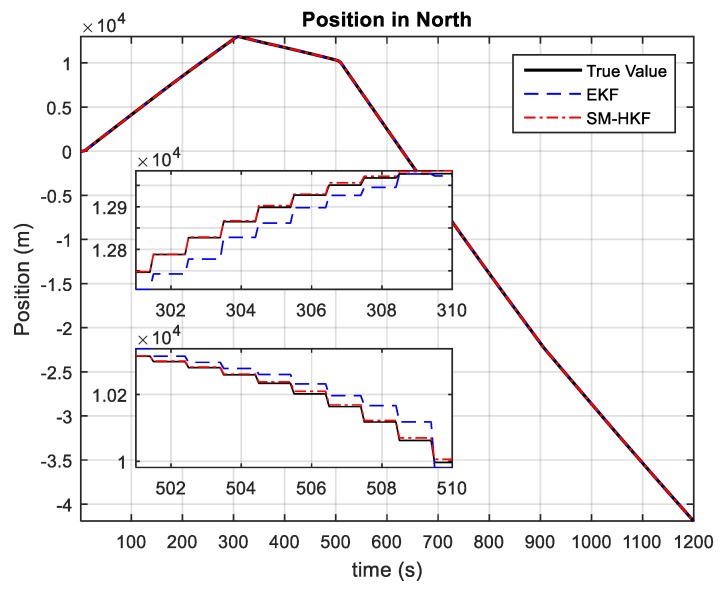
The positions in North estimated by EKF and SM-HKF.

Figure 3 and Figure 4 depict the position estimations of the vehicle achieved by EKF and the proposed SM-HKF. It can be seen that, in the time segments without acceleration variations, that is, without UBB error, both EKF and SM-HKF can estimate the vehicle position in high accuracy because the systematic uncertainty is of a stochastic nature obeying the Gaussian distribution. However, due to its incapability in restraining the effect of UBB error on the filtering solution, EKF has a degraded performance in the presence of acceleration variations. This phenomenon can be observed in the time segments (500 s, 510 s) and (900 s, 910 s) for the position in East as well as in the time segments (300 s, 310 s) and (500 s, 510 s) for the position in North. In contrast, the proposed SM-HKF can track the vehicle position effectively even in the presence of UBB error, since the proposed SM-HKF determines the Kalman gin matrix under the criterion of minimum mean squared error with the consideration of both UBB error and stochastic error.

**Figure 5:**
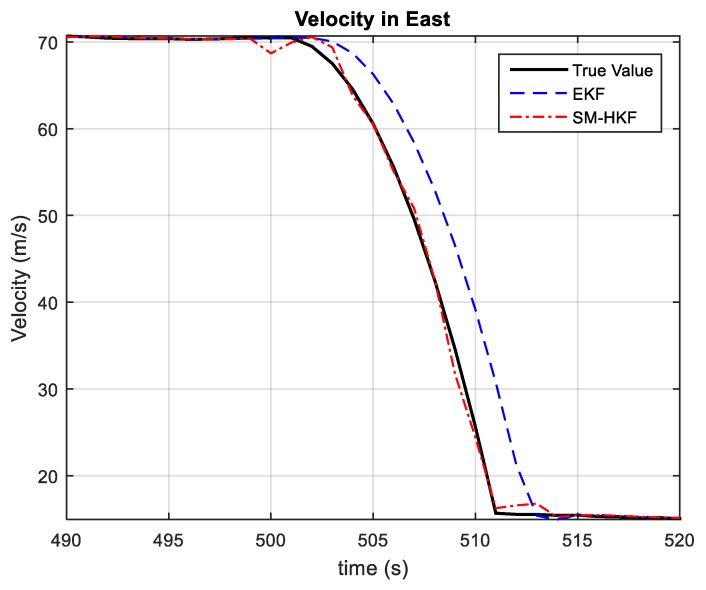
The velocities in East estimated by EKF and SM-HKF from 490 s to 520 s.

**Figure 6:**
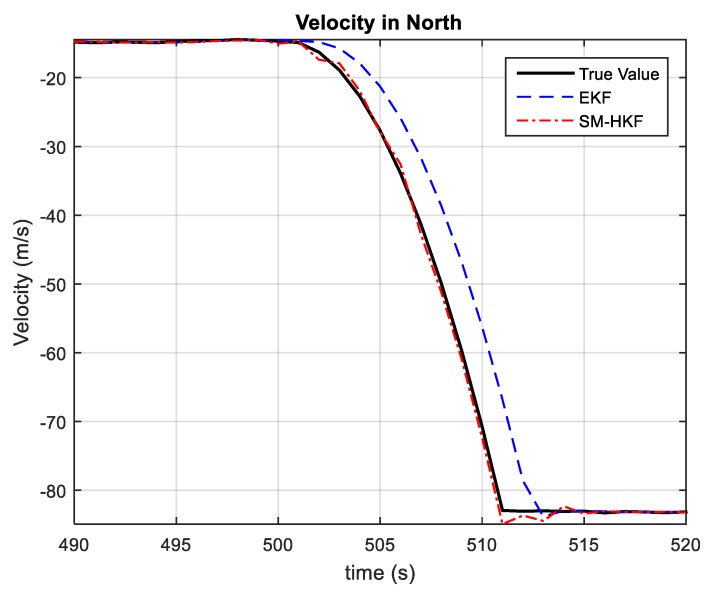
The velocities in North estimated by EKF and SM-HKF from 490 s to 520 s.

Figure 5 and Figure 6 show the vehicle velocities estimated by EKF and SM-HKF in the time segment (490 s, 520 s) involving significant UBB errors, where a similar phenomenon as in Figure 3 and Figure 4 can also be observed. It is easy to verify that EKF results in relatively large estimation biases due to the lack of the robustness against UBB errors. In contrast, since it takes UBB error into account in the Kalman filtering procedure, the vehicle velocity estimated by SM-HKF is much more accurate than that by EKF.

**Figure 7:**
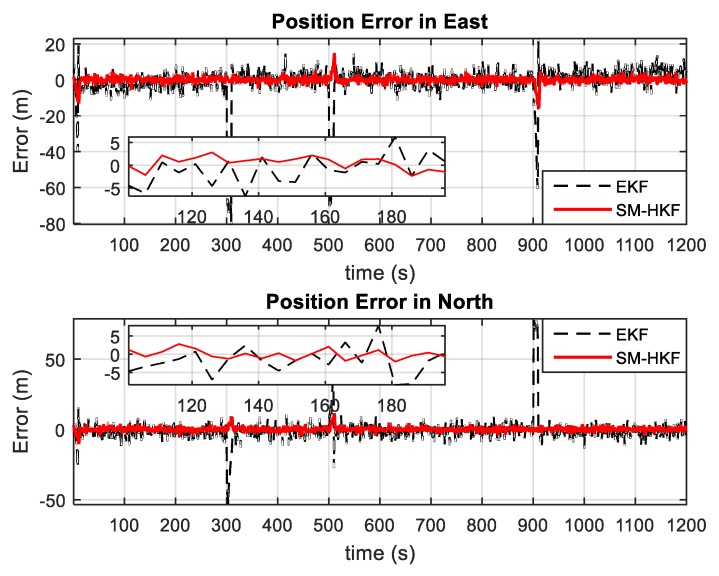
Position errors by EKF and SM-HKF.

**Figure 8:**
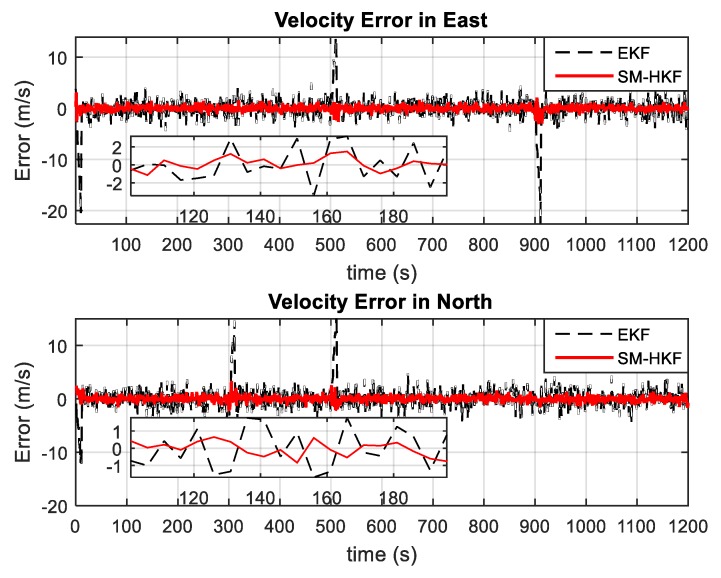
Velocity errors by EKF and SM-HKF.

In addition, by repeating the above simulation 50 times, the Monte Carlo method was also employed to evaluate the SM-HKF robustness comparing to EKF from the statistical perspective. Figure 7 and Figure 8 show the estimation errors in terms of position and velocity obtained by EKF and SM-HKF, respectively. It can be seen that in the time segments without the UBB errors, the estimation error obtained by EKF is slightly larger than that by SM-HKF due to the negligence of the linearization error in the measurement model. However, in the time segments with UBB errors, the difference of estimation error between EKF and SM-HKF become quite evident. As shown in Table 2, the means of the root mean square error (RMSE) of the position errors obtained by SM-HKF are at least 74.4% smaller than those obtained by EKF and the means of the RMSE of the velocity errors by SM-HKF are at least 82.7% smaller than those by EKF. The proposed SM-HKF outperforms EKF significantly due to its capability to resist the influence of UBB error on system state estimation.

The above simulations and analysis demonstrate that the proposed SM-HKF can effectively inhibit the influences of both UBB error and stochastic error on system state estimation by adaptively adjusting the Kalman gain matrix under the criteria of minimum mean squared error, leading to the improved robustness against systematic uncertainty and the higher filtering accuracy than EKF for nonlinear state estimation.

## 5. Conclusions

This paper presents a new SM-HKF to address the issue of nonlinear state estimation with systematic uncertainty. This method derives the optimal Kalman gain to minimize the mean squared error of the state estimate in the framework of KF, leading to the capability in handling both UBB error and stochastic error simultaneously in the filtering procedure. It improves EKF using the concept of set-membership to resist the effect of UBB error on the filtering solution. It also avoids the loss of accuracy in EKF due to the negligence of the linearization error. Further, the proposed SM-HKF incorporates the set-membership estimation in the KF framework, leading to a promising solution for nonlinear state estimation under systematic uncertainty composed of both UBB error and stochastic error. The simulation results and comparison analysis demonstrate the effectiveness and superiority of the proposed SM-HKF in comparison with EKF.

Future research will focus on the in-depth theoretical analysis of the convergence of SM-HKF to facilitate the application of the proposed method in various fields.

## Figures and Tables

**Table 1:** Vehicle acceleration variations.

Time Segment	Vehicle Acceleration Variations	
	East	North
(1–10 s)	Increase: (1.5 m/s^2^)/s	Increase: (1.0 m/s^2^)/s
(301–310 s)	Increase: (0.1 m/s^2^)/s	Decrease: (1.0 m/s^2^)/s
(501–510 s)	Decrease: (1.0 m/s^2^)/s	Decrease: (1.2 m/s^2^)/s
(901–910 s)	Increase (1.5 m/s^2^)/s	Increase: (0.2 m/s^2^)/s
Others	Stochastic fluctuation obeys *N*(0, 0.2^2^)	

**Table 2:** The means of the RMSEs of estimation errors obtained by EKF and SM-HKF during the time segment with unknown but bounded (UBB) errors.

Methods		Positions (m)	Velocities (m/s)
EKF	East	18.30	9.56
	North	16.51	9.17
SM-HKF	East	4.68	1.64
	North	4.04	1.59

## References

[1] Qi J., Sun K., Wang J., Liu H. (2018). Dynamic state estimation for multi-machine power system by unscented Kalman filter with enhanced numerical stability. IEEE Trans. Smart Grid.

[2] Wang Z., Shen X., Zhu Y. Set-membership information fusion for multisensor nonlinear dynamic systems. Proceedings of the 20th International Conference on Information Fusion.

[3] Fernández-Cantí R.M., Blesa J., Puig V., Tornil-Sin S. (2016). Set-membership identification and fault detection using a Bayesian framework. Int. J. Syst. Sci..

[4] Farahmand S., Roumeliotis S.I., Giannakis G.B. (2011). Set-membership constrained particle filter: Distributed adaptation for sensor networks. IEEE Trans. Signal Process..

[5] Hu G., Wang W., Zhong Y., Gao B., Gu C. (2018). A new direct filtering approach to INS/GNSS integration. Aerosp. Sci. Technol..

[6] Hu G.G., Gao S.S., Zhong Y.M. (2015). A derivative UKF for tightly coupled INS/GPS integrated navigation. ISA Trans..

[7] Hu G., Gao S., Zhong Y., Gao B., Subic A. (2015). Modified strong tracking unscented Kalman filter for nonlinear state estimation with process model uncertainty. Int. J. Adapt. Control Signal Process..

[8] Gustafsson F., Hendeby G. (2011). Some relations between extended and unscented Kalman filters. IEEE Trans. Signal Process..

[9] Boutayeb M., Rafaralahy H., Darouach M. (1997). Convergence analysis of the extended Kalman filter used as an observer for nonlinear deterministic discrete-time systems. IEEE Trans. Autom. Control.

[10] Hu G., Ni L., Gao B., Zhu X., Wang W., Zhong Y. (2020). Model predictive based unscented Kalman filter for hypersonic vehicle navigation with INS/GNSS integration. IEEE Access.

[11] Noack B., Pfaff F., Hanebeck U.D. Optimal Kalman gains for combined stochastic and set-membership state estimation. Proceedings of the 51st IEEE Conference on Decision and Control (CDC).

[12] Meinhold R.J., Singpurwalla N.D. (1983). Understanding the Kalman filter. Am. Stat..

[13] Hu G., Gao B., Zhong Y., Ni L., Gu C. (2019). Robust unscented Kalman filtering with measurement error detection for tightly coupled INS/GNSS integration in hypersonic vehicle navigation. IEEE Access.

[14] Zhao Y., Gao S.S., Zhang J., Sun Q.N. (2014). Robust predictive augmented unscented Kalman filter. Int. J. Control Autom. Syst..

[15] Jia B., Xin M., Cheng Y. (2013). High-degree cubature Kalman filter. Automatica.

[16] Chisholm T., Lins R., Givigi S. (2019). FPGA-based Design for Real-time Crack Detection based on Particle Filter. IEEE Trans. Ind. Inform..

[17] Zhao J., Netto M., Mili L. (2016). A robust iterated extended Kalman filter for power system dynamic state estimation. IEEE Trans. Power Syst..

[18] Meng Y., Gao S., Zhong Y., Hu G., Subic A. (2016). Covariance matching based adaptive unscented Kalman filter for direct filtering in INS/GNSS integration. Acta Astronaut..

[19] Meng Y., Gao S., Zhong Y., Hu G., Subic A. (2019). A Robust Cubature Kalman Filter with Abnormal Observations Identification Using the Mahalanobis Distance Criterion for Vehicular INS/GNSS Integration. Sensors.

[20] Zhang Q., Meng X., Zhang S., Wang Y. (2015). Singular value decomposition-based robust cubature Kalman filtering for an integrated GPS/SINS navigation system. J. Navig..

[21] Scholte E., Campbell M.E. (2003). A nonlinear set-membership filter for online applications. Int. J. Robust Nonlinear Control.

[22] Yang F., Li Y. (2009). Set-membership filtering for discrete-time systems with nonlinear equality constraints. IEEE Trans. Autom. Control.

[23] Ge X., Han Q., Wang Z. (2019). A dynamic event-triggered transmission scheme for distributed set-membership estimation over wireless sensor networks. IEEE Trans. Cybern..

[24] Maksarov D., Norton J. (1996). State bounding with ellipsoidal set description of the uncertainty. Int. J. Control.

